# Supplementary material for: MetaRibo-Seq measures translation in microbiomes
Source: Nat Commun. 2020 Jun 29;11:3268. doi: 10.1038/s41467-020-17081-z (PMC7324362; doi:10.1038/s41467-020-17081-z)
Supplement: Supplementary file 10 — Supplementary Data 7 [file 41467_2020_17081_MOESM10_ESM.zip › File2/Confidence_High_Taxonomy/64026_out.krona.html]

Javascript must be enabled to view this page.

members
magnitude
magnitudeUnassigned
count
unassigned
taxon
rank

64026\_out

11

11
2
superkingdom

11
1239
phylum

11
186801
class

186802
order
11

4
186803
family

4
572511
genus


SRS019068\_contig\_number\_17893SRS1041133\_contig\_number\_24569SRS143466\_contig\_number\_15824SRS144362\_contig\_number\_12757
33039
species
4

7
family
541000

genus

SRS018313\_contig\_number\_contig-100\_1583.64342SRS020233\_contig\_number\_16833SRS063190\_contig\_number\_7594SRS063518\_contig\_number\_contig-100\_3990.207257
1263
6
4

species

SRS062654\_contig\_number\_8469
2293146
1


SRS104636\_contig\_number\_23726
2293184
species
1

1

SRS148511\_contig\_number\_13459
1898205
species
